# Supplementary material for: BRD3 Regulates the Inflammatory and Stress Response in Rheumatoid Arthritis Synovial Fibroblasts
Source: Biomedicines. 2023 Nov 30;11(12):3188. doi: 10.3390/biomedicines11123188 (PMC10741099; doi:10.3390/biomedicines11123188)
Supplement: Supplementary file 1 [file biomedicines-11-03188-s001.zip › Supplementary figures.pdf]

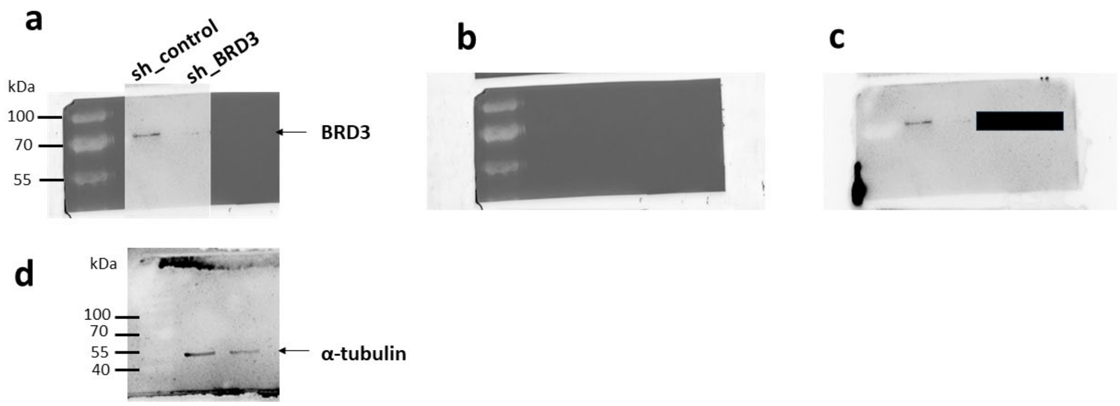

Supplementary Figure S1: Full length Western blot membranes for blots shown in Figure 1. BRD3 was silenced in FLS. Membranes were cut prior probing with the primary antibodies. Protein size markers are shown on the left side of membranes. **(a)** The original size of the membrane for BRD3 is shown in the background of the Western blot image. Images of **(b)** the protein size marker and size of membrane, and **(c)** BRD3 are in shown prior overlaying the images. Black boxes hide bands from samples not discussed in the manuscript. **(d)** The original size of the membrane for α-tubulin is shown. The membrane marker is visible and was therefore not imaged separately.

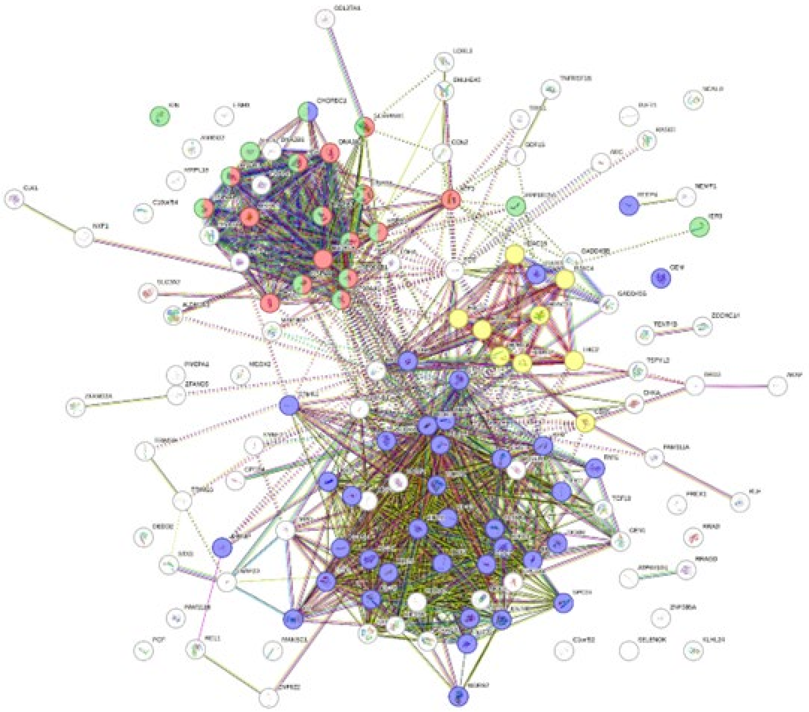

number	type	description	FDR
GO:0006986	GO-term	Response to unfolded protein	7.14×10^{-12}
GO:0022402	GO-term	Cell cycle process	6.44×10^{-16}
CL:4766	Local network cluster (STRING)	Structural constituent of chromatin	3.20×10^{-06}
KW-0364	Annotated Keywords (UniProt)	Stress response	1.04×10^{-14}

Supplementary figure S2: Pathway enrichment analysis using the STRING data base. The analysis is based on 144 overlapping differentially expressed genes in TNF-stimulated and untreated FLS silenced for BRD3, which were identified in Figure 1e. Edges indicate protein-protein interactions. Colors indicate proteins enriched in different pathways which are specified in the table below the graph.

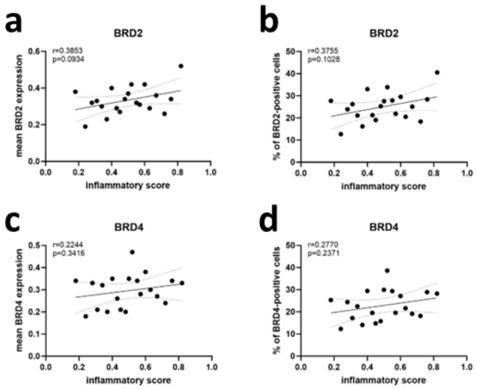

Supplementary figure S3: Correlation of BRD2 and BRD4 with inflammatory scores in synovial tissues. Correlation of **(a)** mean BRD2 expression, **(b)** percentage of BRD2-positive cells, **(c)** mean BRD4 expression and **(d)** percentage of BRD4-positive cells with inflammatory scores of synovial tissues are shown. Data are based on scRNA-seq data sets available at the BroadSingleCellPortal [28].

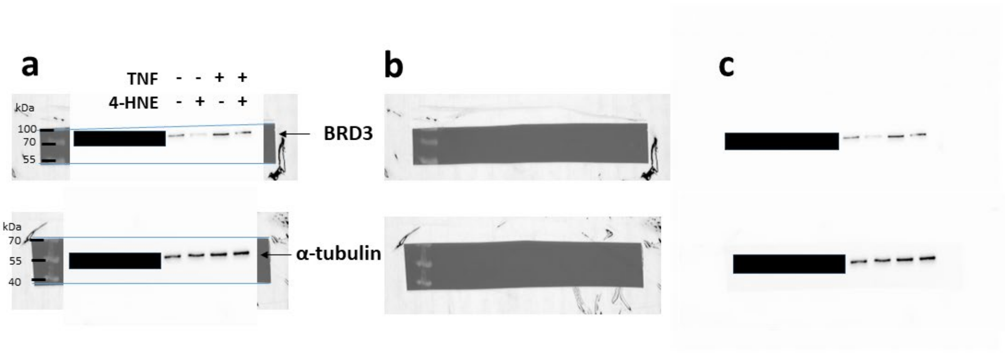

Supplementary Figure S4: Full length Western blot membranes for blots shown in Figure 6. FLS were treated with 4-HNE and TNF, or a combination of both. Membranes were cut prior probing with the primary antibodies. Protein size markers are shown on the left side of membranes. **(a)** The original size of the membranes is shown in the background of the Western blot images. The membrane edges are indicated by blue lines. Images of **(b)** the protein size marker and size of membranes, and **(c)** BRD3 and α-tubulin are in shown prior overlaying the images. Black boxes hide bands from samples not discussed in the manuscript.

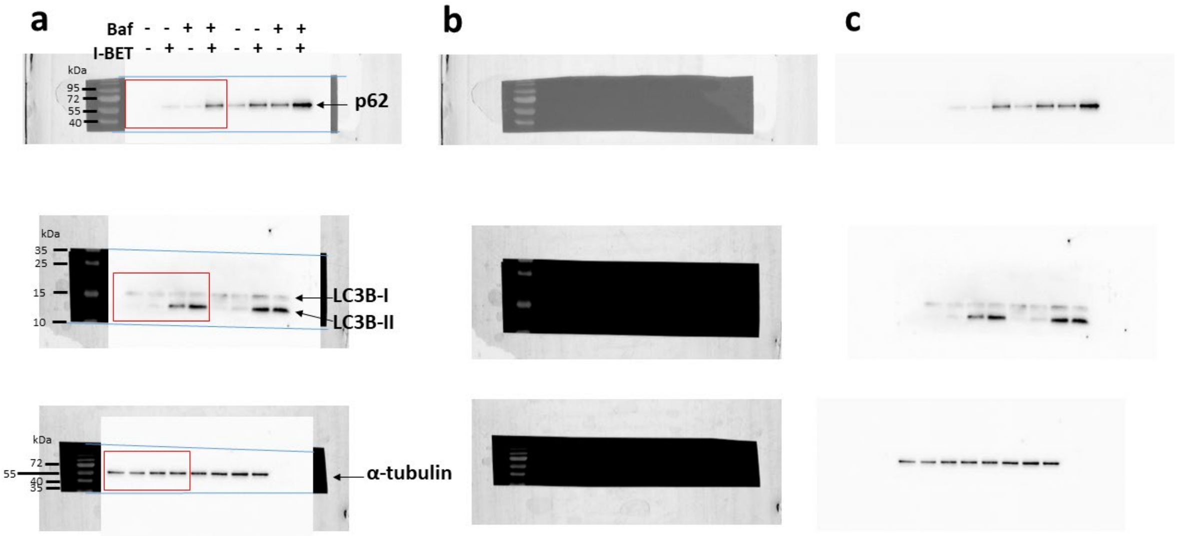

Supplementary Figure S5: Full length Western blot membranes for blots shown in Figure 7. FLS were treated with I-BET in absence and presence of bafilomycin (baf). Membranes were cut prior probing with the primary antibodies. Protein size markers are shown on the left side of membranes. **(a)** The original size of the membranes is shown in the background of the Western blot images. The membrane edges are indicated by blue lines. Red boxes indicate the samples shown in the main manuscript. Images of **(b)** the protein size marker and size of membranes, and **(c)** p62, LC3B and α-tubulin are in shown prior overlaying the images.
